# Society for *Molecular Biology and Evolution*, Council and Business Meetings, 2017, Austin, TX

**DOI:** 10.1093/molbev/msx296

**Published:** 2017-12-22

**Authors:** David D Pollock

**Affiliations:** Department of Biochemistry and Molecular Genetics, University of Colorado School of Medicine, Aurora, CO

## Council Meeting

### 1. Call to Order

President Laura Landweber called the council meeting to order at 8:30 AM, Sunday, July 2, 2017. In attendance were President Laura Landweber, Past-President George Zhang, President-Elect Bill Martin, Councilors Emma Teeling, Kateryna Makova, Maud Tenaillon, Adam Eyre-Walker, Joanna Masel, Jay Storz, Secretary David Pollock, Treasurer Juliette de Meaux, and MBE Editor-in-Chief Sudhir Kumar (*ex officio*).

#### Non-Council Members in Attendance

Cathy Kennedy. Claus Wilke (Austin—SMBE2017), Koichiro Tamura (Yokohama—SMBE2018), Yoko Satta (Yokohama—SMBE2018), James McInerney (Manchester—SMBE2019) attended part of the meeting, an Oxford University Press delegation consisting of Alex Beaumont and Adam Leary attended part of the meeting, and Terry Leatherman (Allen Press) attended part of the meeting.

### 2. Approval of Minutes

The council approved the minutes of the 2016 Council and Business meeting, published in the January 2017 issue of MBE (first published online: December 29, 2016).

### 3. Treasurer’s Report

Treasurer Juliette de Meaux summarized the financial state of the Society, with details on journal and meeting revenue and expenses. The records show that the Society is in excellent financial health, with total net assets of US $3, 517, 858. More details can be found in the Treasurer’s report.

### 4. Secretary’s Report

Secretary David Pollock reviewed the council actions over the preceding year. Central issues were interactions and contracts with MBE and GBE publisher OUP, Allen Press, and MCI, as well as the upcoming GBE Editor-in-Chief replacement and diversity issues in a number of areas; details are summarized below.

### 5. Election of SMBE Officers

The nominations committee for nominating candidates for the position of President-elect, for Treasurer, and for two council positions was chaired by Wen-Hsiung Li (Chicago). The members of the committee were Ruth Hershberg (Haifa, Israel), Marc Robinson-Rechavi (Lausanne, France), Beth Shapiro (Santa Cruz), Gunter Wagner (Yale), and David Pollock SMBE Secretary, (Denver), *ex officio*, as per by-laws. The membership of the society was informed by email of the committee composition in early 2017 and was asked to nominate candidates for the various positions.

The nominees for President-elect were Anne Stone (Arizona State University) and Aoife McLysaght (Trinity University, Ireland). The nominees for Treasurer were Jeff Thorne (North Carolina State University) and Michael Rosenberg (Arizona State University).

The nominees for the councilor positions were Belinda Chang (University of Toronto, Canada), Keith Crandall (George Washington University), Nicholas Galtier (Université Montpellier, France), and Anthony Poole (University of Canterbury, New Zealand).

The membership of the society was issued online ballot details through Allen Press with the voting period open from April 21 to June 1, 2017. The results of the ballot are that the President-elect is Aoife McLysaght, the Treasurer is Jeff Thorne and the newly elected councilors are Belinda Chang and Nicholas Galtier.

The council does and will continue to remind the nominating committee every year of gender and geographic diversity goals, but maintains flexibility in how this is achieved by the committee.

### 6. Editors’ Reports

The Editors in Chief of MBE and GBE gave reports on the current state of their journals (see separate editors’ reports). There is a healthy submission rate for both journals, with respective acceptance rates of 25% and 50%, and both journals are working to improve or maintain gender and geographic balance on the editorial boards. OUP also gave a presentation on their work on the journals and transition to a new online publishing system.

### 7. Other Activities

#### i) Future Annual Meetings

The next annual meeting of the society will be held from July 8–12 in the Pacifico Yokohama, Yokohama, Japan. The 2019 meeting will be held in Manchester, United Kingdom, and by vote it was decided that the 2020 Annual meeting will be held in Quebec City, Canada.

#### ii) Satellite Meetings and Interdisciplinary (Regional) Meetings

Councilor Kateryna Makova organized review of satellite meeting proposals and councilor Maud Tenaillon organized review of interdisciplinary meeting proposals. It was voted to fund proposed interdisciplinary (regional) meetings in Lyon, France (“Interdisciplinary approach for molecular evolution”), Cali, Colombia (“Colombian association for evolutionary biology”), and Pittsburgh, Pennsylvania (“Three Rivers Evolution Event”). It was voted to fund satellite meetings in Utah (“Molecular evolution and the cell”), India (“Evolution of microbes in natural and experimental populations—synthesis and synergies”), Rhode Island (“Modern methods for the study of ancient DNA”), Japan (“Genome evolution in pathogen transmission and disease”), and Italy (“Evolution of genome architecture”). It was also voted to fund a special symposium in Pennsylvania (“Molecular evolutionary genetics”) to honor one of SMBE’s founders, Masatoshi Nei.

#### iii) Undergraduate Mentoring and Diversity Program

Councilor Joanna Masel organized the undergraduate mentoring and diversity program at the annual meeting. This program was able to make 11 awards. By acclamation of the council, it was decided that in the future, senior faculty can also be mentors.

#### iv) Allan Wilson Junior Award for Independent Research

This award is given to outstanding members of the SMBE community who are in the early stages of an independent research career. The 2017 recipient was Mia Levine (University of Pennsylvania).

#### v) Margaret Dayhoff Mid-Career Award

This award is intended for outstanding members of the SMBE community who are in the midst of their research careers. The 2017 recipient was Toni Gabaldón, (Center for Genomic Regulation, Spain).

#### vi) Motoo Kimura Lifetime Achievement Award

This award is intended for outstanding senior members of the SMBE community. The 2017 recipient was W. Ford Doolittle (Dalhousie University, Canada).

#### vii) Community Service Award

This award is intended for members of SMBE who have provided exceptional service to SMBE and the broader scientific community. The 2017 recipient was Sudhir Kumar (Temple University).

#### viii) Poster Award Recipients

President-elect Bill Martin ran the award process with 24 people assisting. There were about 800 posters, including about 350 undergraduates, 200 postdoctoral researchers, and 29 undergraduate students. Motions passed unanimously to allow up to 9 poster awards at discretion of organizers, and increase award to $500 at all three levels. The 2017 postdoctoral best poster awards went to Marc Tollis (Arizona State University), Elizabeth Atkinson (Stony Brook University), and Atahualpa Castillo Morales (University of Bath, UK). The best poster for graduate students went to James Fleming (University of Bristol, UK), Pinglin Cao (Tohoku University, Japan), and Magdalena Kubiak (Adam Mickiewicz University, Poland). The best poster for undergraduate students went to Isabela Jeronimo Bezerra Marcos (Universidade Federal da Paraíba, Brazil), Joseph Palmer (University of Sheffield, UK), and Dan Werndly (Curtin University, Australia).

#### ix) Fitch Prize Winner and Graduate/Postdoctoral Trav Awards

Past-president George Zhang organized the Fitch speaker and travel award judging with a committee of five councilors. 358 people applied for some combination of the Fitch Prize, travel and registration awards, and there were 8 Fitch finalists, 52 travel awards, and 93 registration awards. The Fitch Prize winner was Anna Vickrey (University of Utah) for a talk titled “Domestic pigeon’s checkered past: wing color pattern variation is associated with one gene, two mechanisms, and interspecific introgression”.

#### x) Best Graduate Student Paper Awards

The MBE best paper award was given to Alejandra Rodríguez-Verdugo (Uppsala University, Sweden) for the paper “First-step mutations during adaptation restore the expression of hundreds of genes”, and the GBE best paper award was given to Anouk Willemsen (MIVEGC—Centre IRD de Montpellier, France) for the paper “Predicting the stability of homologous gene duplications in a plant RNA virus”.

#### xi) Childcare Awards

There were 33 childcare awards provided, in addition to on-site childcare, with children ranging from 1 month to 11 years old.

#### xii) Editor-in-Chief of Genome Biology and Evolution

The council unanimously approved that, if funds are available when an editor-in-chief of a successful SMBE journal steps down, we will, in principle, provide US $75, 000 per full term served as editor-in-chief to support the research program of the former editor-in-chief. This amount may be adjusted for inflation or for circumstances such as starting a new successful journal. Term limits for editors in chief were discussed but not implemented at this time, although this issue may be revisited in the near future.

#### xiii) Sexual Harassment Policy

It was determined that the Sexual Harassment Policy approved for the Austin meeting should be kept as permanent policy.

#### xiv) Contracts with Allen Press, MCI, and OUP

The council made progress in negotiating a contract renewal with Allen Press for web and email services, and a multi-year contract with MCI for meeting organization services.

#### xv) Sharing Gender Statistics of SMBE Members

It was unanimously agreed that SMBE would share gender statistics of SMBE members, as requested by Nicolas Rode, but only the total numbers. Councilor Maud Tenaillon gave a presentation on estimated gender and career stage distributions at recent SMBE meetings (separate publication).

#### xvi) New Journals

The idea of a new journal was discussed and it was determined that there is no need to start a new journal at this time. It was also discussed whether SMBE should support the new-style journal *Peer Community In Evolutionary Biology (PCI Evol Biol)*, and partly due to the ambiguity of their “peer-reviewed” status, it was decided that they should not use the SMBE logo and SMBE will not be providing support at this time, although we wish them good luck in their efforts.

## Business Meeting

### 1. Call to Order

President Laura Landweber called the council meeting to order at 12 PM on July 4.

### 2. Business

The president summarized the discussion at the council meetings, announced the location of the 2018, 2019, and 2020 meetings, announced the outcomes of the elections and congratulated the organizers of the Austin meeting. Koichiro Tamura gave a short presentation on the logistics of the 2018 annual meeting in Yokohama, Japan.

